# Rhusflavone Modulates Osteoclastogenesis Through RANKL-Induced AKT Signaling in Bone Marrow-Derived Macrophages

**DOI:** 10.3390/ijms26073025

**Published:** 2025-03-26

**Authors:** Hyung-Mun Yun, Bomi Kim, Eonmi Kim, Kyung-Ran Park

**Affiliations:** 1 Department of Oral and Maxillofacial Pathology, School of Dentistry, Kyung Hee University, Seoul 02447, Republic of Korea; 2 National Institute for Korean Medicine Development, Gyeongsan 38540, Republic of Korea; bom0203@nikom.or.kr (B.K.); minnie60@nikom.or.kr (E.K.); 3 Honam Regional Center, Korea Basic Science Institute (KBSI), Gwangju 61751, Republic of Korea

**Keywords:** Rhusflavone, RANKL, osteoclast, bone, BMM, AKT

## Abstract

Osteoclast differentiation inhibition is a viable treatment strategy for osteoporosis because osteoclasts play a vital role in disease progression. Rhusflavone (Rhus), a biflavonoid, exhibits a sedative–hypnotic effect via the positive allosteric modulation of GABA(A) receptors. Although several biflavonoids possess activities that help prevent bone loss, the potential effects of Rhus on osteoclastogenesis have not been reported yet. In this study, we investigated the effects and underlying biological mechanisms of Rhus isolated from the dried roots of *Rhus succedanea* on osteoclastogenesis in primary cultured bone marrow-derived macrophages. No cytotoxicity was observed in bone marrow macrophages (BMMs) or during osteoclast differentiation. However, Rhus reduced the number of tartrate-resistant acid phosphatase (TRAP)-positive multinuclear osteoclasts during receptor activator of nuclear factor kappa B ligand (RANKL)-induced osteoclastogenesis. The results of F-actin ring formation demonstrated that Rhus suppresses the bone resorption activity of osteoclasts. Additionally, Rhus inhibits the expression of osteoclast differentiation marker proteins, specifically c-Fos and NF-ATc1. Western blot analysis revealed that Rhus primarily attenuated RANKL-mediated key signaling pathways, particularly the AKT signaling pathway. Furthermore, we found that the AKT activator and inhibitor pharmacologically abolished and enhanced the inhibitory effects of Rhus on osteoclast differentiation, respectively. Taken together, our findings provide evidence that Rhus is a promising biologically active compound that regulates osteoclast differentiation by inhibiting the AKT signaling pathway, which may contribute to future drug development.

## 1. Introduction

Osteoporosis is a prevalent, chronic, and systemic bone disease characterized by significant bone mass loss, which increases a patient’s vulnerability and leads to compromised bone strength and high fracture risk [1,2]. One critical issue is the complicated multistep process known as osteoclast differentiation or osteoclastogenesis committed by hematopoietic precursors and macrophages, which play a critical role in regulating skeletal integrity and structure [3]. The key signaling pathway for osteoclastogenesis is initiated by two cytokines from the tumor necrosis factor (TNF) family: macrophage colony-stimulating factor (M-CSF) and receptor activator of nuclear factor kappa B ligand (RANKL) [4]. The released RANKL interacts with its receptor RANK expressed on hematopoietic precursor and macrophage surface [5]. The interaction between RANKL and RANK recruits TNF receptor-associated factor (TRAF) family proteins, including TRAF2 and TRAF6, and activates various signaling pathways leading to the expression of nuclear factor of activated T-cell cytoplasmic 1 (NF-ATc1) and c-Fos transcription factors, which are important for osteoclast differentiation [5,6,7]. However, bone stability is disrupted by excess bone resorption through increased osteoclast differentiation and activity, ultimately resulting in chronic osteolysis and contributing to bone diseases, such as osteoporosis [8]. Therefore, identifying compounds that can regulate osteoclastogenesis and osteoclast activity is essential to effectively treat osteoporosis. Clinically, anti-catabolic drugs, such as bisphosphonates, estrogen replacement therapy, and RANKL inhibitors, are widely used to inhibit osteoclastogenesis and increase bone mass [9,10]. However, the adverse effects of anti-catabolic drugs, including an increased risk of post-menopausal breast cancer, endometrial cancer, heart disease, and bisphosphonate-related osteonecrosis of the jaw, present additional challenges for osteoporosis management [11,12,13]. Thus, there is an urgent need to identify effective and safe compounds for treating bone diseases such as osteoporosis.

*Rhus succedanea*, also known as *Toxicodendron succedaneum* L., belongs to the family Anacardiaceae and is found throughout Asia. It has been traditionally used to treat various conditions, including infections, tuberculosis, gum bleeding, asthma, and stress [14,15]. Bioactive compounds such as urushiol, tannins, biflavonoids, and phenols have been isolated from *Rhus succedanea* [14,15]. Rhusflavone (Rhus) is a natural biflavonoid primarily isolated from *Rhus succedanea* [16]. Rhus leads to sleep via positive allosteric modulation of GABA(A) receptors [17]. Previous studies have demonstrated that several biflavonoid compounds, including amentoflavone, sciadopitysin, and ginkgetin, suppress osteoclastogenesis to prevent bone loss [18,19,20]. Amentoflavone prevents osteoclastogenesis by inhibiting NF-κB and MAPK signaling. Sciadopitysin inhibits osteoclast differentiation by targeting NF-κB activation, without altering MAPK phosphorylation. Ginkgetin attenuates osteoclast differentiation by targeting the NF-κB signaling pathway [18,19,20]. Although we isolated and identified Rhus from the dried roots of *Rhus succedanea*, its pharmacological activities on osteoclasts have not yet been determined. Therefore, we investigated the effects of Rhus on osteoclastogenesis. In this study, we demonstrate the potential effects of Rhus on osteoclast differentiation and bone resorption activity, as well as explore the signaling pathways associated with Rhus using primary bone marrow macrophages (BMMs).

## 2. Results

### 2.1. Extractoin and Characterization of Rhus and Its Cytotoxicity

**Figure 1:**
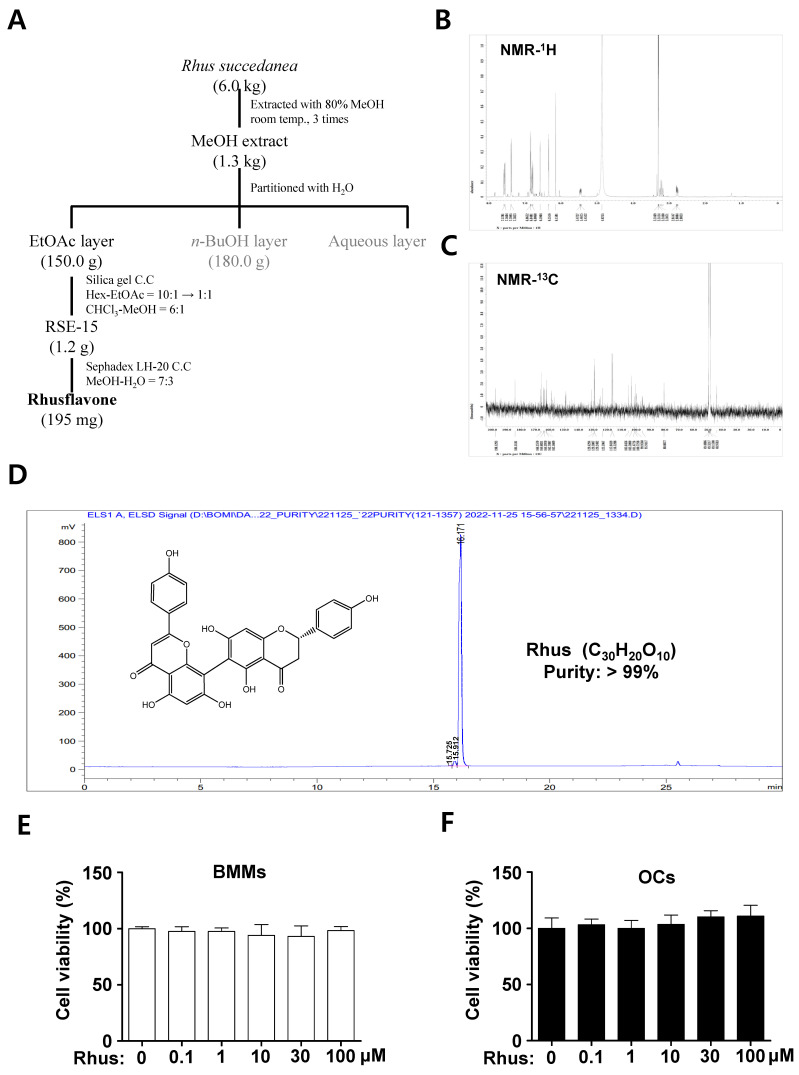
Extractoin of Rhusflavone (Rhus) from the dried roots of *Rhus succedanean* and its cytotoxicity effects. (**A**) Procedure for the isolation of Rhus. (**B**,**C**) ^1^H-NMR (500 MHz, CD3OD) spectrum (**B**) and ^13^C-NMR (125 MHz, CD3OD) spectrum (**C**) of Rhus. (**D**) HPLC analysis of the isolated Rhus. The inset shows the chemical structure, purity, and molecular formula. (**E**,**F**) Bone marrow macrophages (BMMs) were seeded onto 96-well plates and treated with the indicated Rhus concentration for 24 h (**E**). The BMMs were differentiated into osteoclasts using 30 ng/mL macrophage colony-stimulating factor (M-CSF) and 100 ng/mL receptor activator of nuclear factor kappa B ligand (RANKL) for 3 days, and the cells were subsequently treated with the indicated Rhus concentration (**F**). Cell viability was assessed using the 3-(4,5-dimethylthiazol-2-yl)-2,5-diphenyl tetrazolium bromide (MTT) assay. OCs: osteoclasts. The data presented are derived from three separate experiments and are expressed as the mean ± standard deviation (SD).

Rhus (white powder, 195 mg), a biflavonoid, was isolated from dried *R. succedanea* roots as described in Figure 1A and identified using nuclear magnetic resonance (NMR) analysis (Figure 1B,C). The ^1^H-NMR (500 MHz, CD3OD) spectrum displayed peaks at δ 7.58 (^2^H, dd, J = 11.2, 8.9 Hz, H-2′′′, H-6′′′), 7.39 (^2^H, d, J = 8.6 Hz, H-2′, H-6′), 6.86 (^2^H, dd, J = 2.0, 6.6 Hz, H-3′, H-5′), 6.76 (^2^H, dd, J = 8.9, 12.4 Hz, H-3′′′, H-5′′′), 6.59 (^1^H, s, H-3′′), 6.35 (^1^H, d, J = 1.4 Hz, H-6′′), 6.16 (^1^H, s, H-8), 5.47 (^1^H, m, H-2), 3.24 (^1^H, m, H-3b), and 2.80 (^1^H, m, H-3a) (Figure 1B). The ^13^C-NMR (125 MHz, CD3OD) spectrum displayed peaks at δ 198.1 (C-4), 184.5 (C-4′′), 166.8 (C-7), 166.3 (C-2′′), 164.7 (C-9), 164.4 (C-7′′), 164.3 (C-5), 163.8 (C-5′′), 162.7 (C-4′′′), 162.6 (C-4′), 159.3 (C-9′′), 131.2 (C-1′), 129.4 (C-2′′′), 129.3 (C-6′′′), 129.2 (C-2′, C-6′), 123.6 (C-1′′′), 117.0 (C-3′′′, C-5′′′), 116.5 (C-3′, C-5′), 105.6 (C-10′′), 103.5 (C-6), 103.2 (C-3′′), 101.5 (C-10), 100.7 (C-8′′), 100.0 (C-6′′), 95.9 (C-8), 80.8 (C-2), and 44.3 (C-3) (Figure 1C). The high-performance liquid chromatography (HPLC) chromatogram and chemical structure of Rhus (chemical formula C_30_H_20_O_10_, >99% pure) are shown in Figure 1D. The cytotoxicity of Rhus was initially assessed to investigate its potential effects on osteoclastogenesis. The 3-(4,5-dimethylthiazol-2-yl)-2,5-diphenyl tetrazolium bromide (MTT) experiment revealed no cytotoxic effects of 1–100 μM Rhus in the primary BMMs derived from mice bone marrow cells (Figure 1E) or under osteoclast differentiation (Figure 1F). These findings suggest that Rhus does not induce cytotoxicity in primary cultured BMMs or during RANKL-induced osteoclastogenesis.

### 2.2. Rhus Inhibits the Formation of RANKL-Induced TRAP (+) MNCs and F-Actin Rings

**Figure 2:**
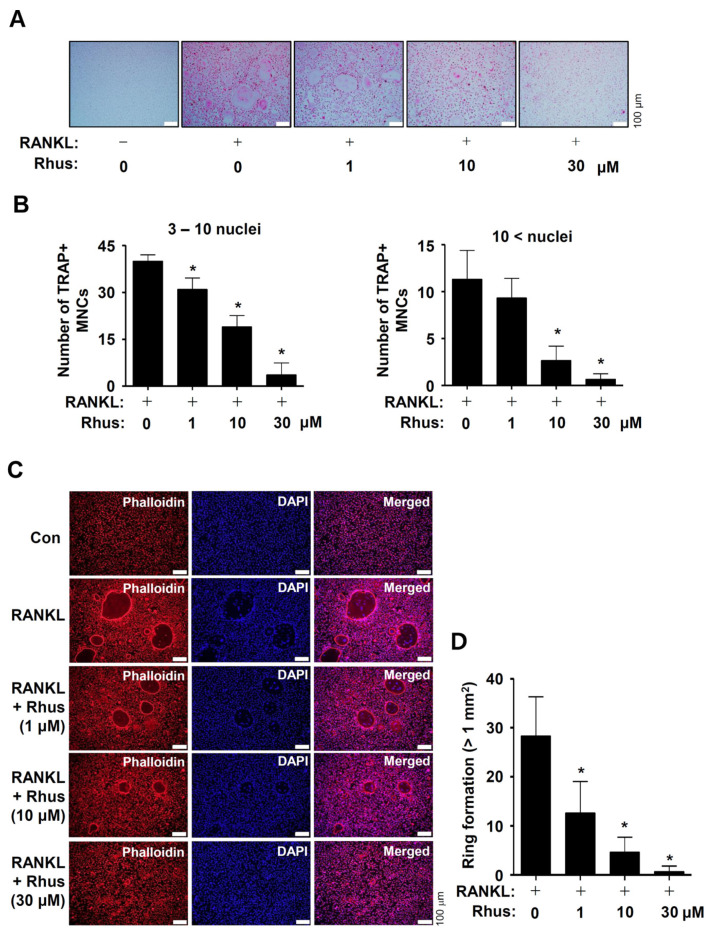
Effects of Rhusflavone (Rhus) on osteoclastogenesis and bone-resorbing activity. (**A**,**B**) Bone marrow macrophages (BMMs) were seeded into 48-well plates and cultured for 5 days in a medium containing 100 ng/mL of receptor activator of nuclear factor kappa B ligand (RANKL) and 30 ng/mL of macrophage colony-stimulating factor (M-CSF) with 1–30 μM of Rhus. Mature osteoclasts were detected with tartrate-resistant acid phosphatase (TRAP) staining (**A**). TRAP-positive multinucleated osteoclasts (MNCs) with over three nuclei were considered as mature osteoclasts and counted under a microscope (**B**). A quantity of 3–10 nuclei (**left**), 10< nuclei (**right**). Scale bar: 100 μm. (**C**,**D**) F-actin ring (red) formation was detected using a fluorescence microscope after staining with TRITC–phalloidin (red) and DAPI (blue) (**C**). The number of ring formations greater than 1 mm^2^ was analyzed (**D**). Scale bar: 100 μm. *, *p* < 0.05 compared to RANKL treatment alone. The data presented are derived from three separate experiments and are expressed as the mean ± standard deviation (SD).

To investigate the potential activities of Rhus on osteoclast differentiation, the BMMs were differentiated by RANKL and M-CSF in the presence or absence of 1–30 μM Rhus. Osteoclastogenesis was analyzed using tartrate-resistant acid phosphatase (TRAP) staining. Rhus dose-dependently suppressed both TRAP-positive staining and TRAP-positive multinucleated osteoclast (MNC) formation (Figure 2A,B). Additionally, Rhus reduced the number of giant osteoclasts compared to RANKL (Figure 2A,B). TRAP is a distinctive marker of osteoclast differentiation that is highly expressed during bone resorption in mature osteoclasts. To demonstrate the inhibitory effect of Rhus on mature osteoclasts, we investigated its effects on osteoclastic bone resorption using an F-actin ring formation assay. Rhus treatment dose-dependently impaired mature F-actin ring formation (Figure 2C,D). Compared with the RANKL-treated osteoclasts, Rhus-treated osteoclasts exhibited fewer and smaller F-actin rings (Figure 2C,D). These results suggest that Rhus suppressed osteoclastogenesis and the ability of mature osteoclasts to resorb bone.

### 2.3. Rhus Suppresses RANKL-Induced Osteoclast-Specific Transcription Factors Induced by RANKL

**Figure 3:**
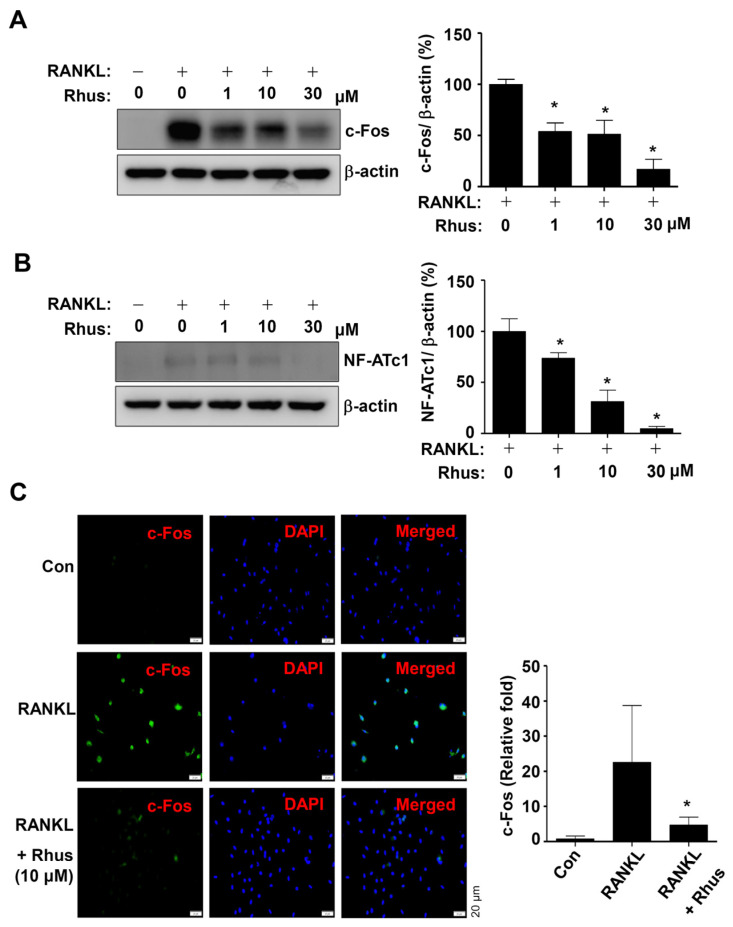
Effects of Rhusflavone (Rhus) on osteoclast-specific transcription factors. (**A**,**B**) Bone marrow macrophages (BMMs) were seeded in 6-well plates and differentiated for 1 day (**A**) or 2 days (**B**). Western blot analysis was used to assess c-Fos and β-actin (**A**); NF-ATc1 and β-actin (**B**) expression levels. The relative level (%) is presented as a bar graph. (**C**) After treating Rhus for 24 h, c-Fos (green) was immunostained. Subsequently, DAPI (blue) was used to stain the nucleus. Relative fluorescence intensity fold changes are presented as bar graphs. *, *p* < 0.05 compared to RANKL treatment alone. The data presented are derived from three separate experiments and are expressed as the mean ± standard deviation (SD).

The effects of Rhus on RANKL-induced osteoclast-specific transcription factors c-Fos and NFATc1 were examined to better understand the mechanisms underlying its inhibitory effects on osteoclastogenesis. The BMMs were differentiated using RANKL for 1 or 2 days in the presence or absence of Rhus. Rhus treatment dose-dependently downregulated c-Fos and NF-ATc1 expression levels compared to RANKL treatment (Figure 3A,B). Furthermore, immunofluorescence analyses confirmed that c-Fos was significantly induced in the RANKL-treated group, whereas c-Fos induction was blocked by treatment with Rhus (Figure 3C). These results suggest that Rhus inhibits osteoclastogenesis by inhibiting osteoclast-specific transcription factors.

### 2.4. Rhus Suppresses RANKL-Induced Intracellular Signaling Pathways

**Figure 4:**
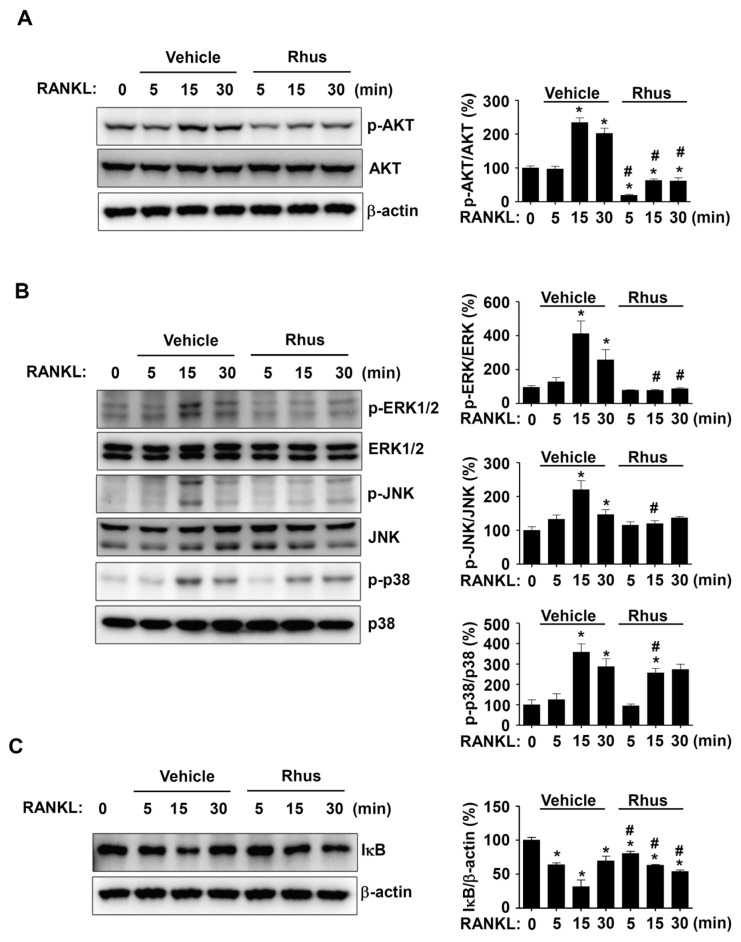
Effects of Rhusflavone (Rhus) on receptor activator of nuclear factor kappa B ligand (RANKL)-induced intracellular signaling pathways. (**A**–**C**) Bone marrow macrophages (BMMs) were seeded on 6-well plates and stimulated in 100 ng/mL RANKL with 10 μM Rhus for the indicated time. AKT, p-AKT, and β-actin (**A**); ERK, p-ERK, JNK, p-JNK, p38, and p-p38 (**B)**; IkB and β-actin (**C**) expression were investigated using western blot analysis. The relative level (%) is presented as a bar graph. *, *p* < 0.05 compared to RANKL treatment alone at 0 min. #, *p* < 0.05 compared between RANKL alone and RANKL + Rhus at each min time point. The data presented are derived from three separate experiments and are expressed as the mean ± standard deviation (SD).

We investigated RANKL-induced signaling proteins involved in osteoclastogenesis to gain a deeper understanding of the mechanisms underlying Rhus’s inhibitory effects on osteoclastogenesis. In the presence or absence of Rhus, the BMMs were treated with RANKL for 0, 5, 15, and 30 min. The signaling proteins AKT, MAPKs, and NF-κB were evaluated using western blot analysis. Rhus significantly and time-dependently reduced AKT activation, as evidenced by decreased AKT phosphorylation at 5, 15, and 30 min (Figure 4A). Additionally, Rhus suppressed MAPK signaling pathways, including ERK1/2, JNK, and p38 (Figure 4B). At 5 and 15 min, IκB was stabilized by Rhus; however, IκB expression decreased at 30 min (Figure 4C). These findings suggest that the inhibitory effects of Rhus on osteoclastogenesis involve RANKL-induced signaling pathways.

### 2.5. Rhus Inhibits Osteoclastogenesis via Suppressing AKT Activation

**Figure 5:**
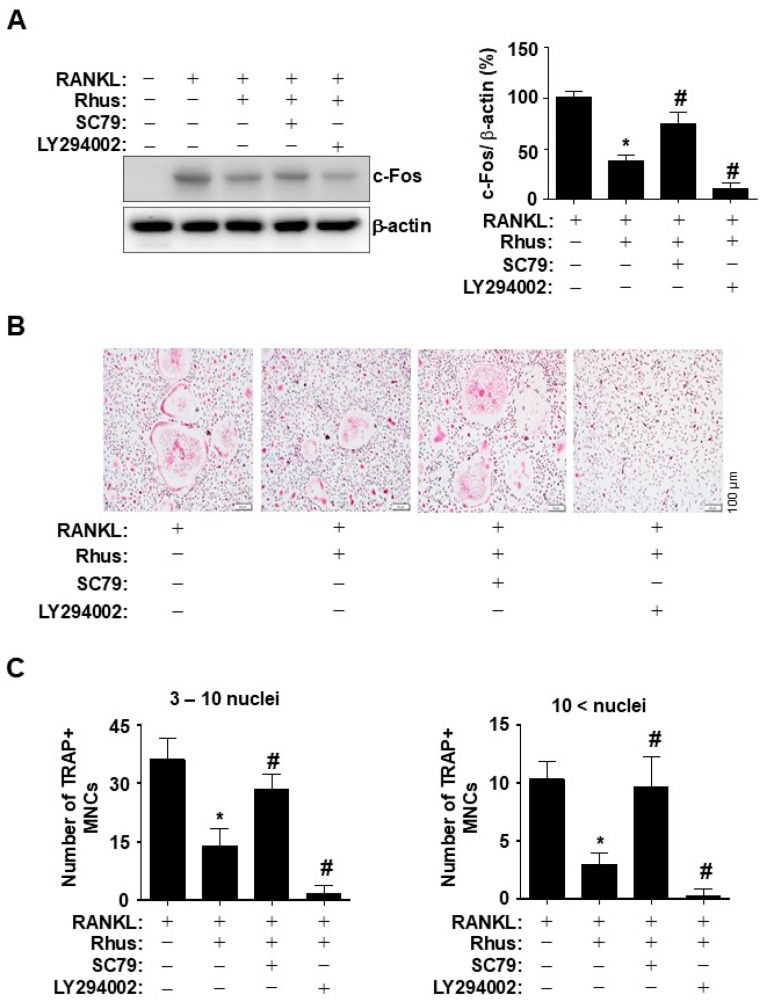
Effects of Rhus on the inhibition of receptor activator of nuclear factor kappa B ligand (RANKL)-induced AKT signaling pathway in osteoclastogenesis. (**A**) Bone marrow macrophages (BMMs) were differentiated with 10 μM Rhus, in the presence or absence of 1 μM SC79 or LY294002, for 1 day. Western blot analysis was used to assess c-Fos and β-actin expression levels. The relative level (%) is presented as a bar graph. (**B**,**C**) The BMMs were cultured in 30 ng/mL M-CSF and 100 ng/mL RANKL with 10 μM Rhus, in the presence or absence of 1 μM SC79 or LY294002, for 5 days. Mature osteoclasts were detected with tartrate-resistant acid phosphatase (TRAP) staining (pink) (**B**). TRAP-positive multinucleated osteoclasts (MNCs) were counted under a microscope (**C**). A quantity of 3–10 nuclei (**left**), 10 < nuclei (**right**). Scale bar: 100 μm. *, *p* < 0.05 compared with RANKL alone. #, *p* < 0.05 compared with RANKL + Rhus. The data presented are derived from three separate experiments and are expressed as the mean ± standard deviation (SD).

To investigate the direct role of Rhus-mediated AKT inhibition during osteoclastogenesis, BMMs were differentiated for 1 d using RANKL in the presence or absence of an AKT activator or inhibitor. Pharmacological AKT activation by SC79 and inhibition by LY294002 blocked and synergistically enhanced the inhibitory effects of Rhus on c-Fos induction, respectively (Figure 5A). To verify the inhibitory effects of Rhus on osteoclastogenesis via the AKT signaling pathway, BMMs were differentiated for 5 days using RANKL in the presence or absence of an AKT activator or inhibitor. Treatment with an AKT activator and inhibitor mitigated and enhanced the Rhus-induced reduction in TRAP-positive MNCs, respectively (Figure 5B). The pharmacological effects were confirmed by analyzing the proportion of TRAP-positive MNCs compared to the number of nuclei (Figure 5C). These findings suggested that the inhibitory effects of Rhus on osteoclastogenesis were mediated through the inhibition of AKT signaling pathways associated with RANKL-induced osteoclast differentiation.

## 3. Discussion

Osteoclasts are multinucleated cells responsible for the resorption of mineralized bone tissue, a process that is essential for both morphogenesis and remodeling [21]. Pathological bone loss occurs due to excessive bone resorption by osteoclasts, which is a consequence of abnormal osteoclastogenesis [3,22,23]. Osteoporosis, a serious public health problem, is a bone disease characterized by low bone mineral density and structural degeneration of the bone tissue, leading to fragility and fracture susceptibility [2]. Bone composition varies seasonally. Seasonal variations influence the levels of growth hormones, which are crucial for regulating bone mass and preventing fractures in patients with osteoporosis [24,25,26,27,28]. Osteoporosis has a significant hormonal component that must be examined from multiple perspectives. The effects of Rhus, a biflavonoid compound isolated from the dried roots of *R. succedanea*, a well-known traditional Chinese medicine, on osteoclastogenesis and its biological mechanism were identified and demonstrated in this study. We found that Rhus suppressed multinucleation, the bone-resorbing activity of osteoclasts, and osteoclast-specific proteins during the osteoclastogenesis of BMMs. Unlike the three existing biflavonoid compounds, amentoflavone, sciadopitysin, and ginkgetin [18,19,20], this study demonstrated that Rhus directly attenuates osteoclastogenesis through the RANKL-induced AKT signaling pathway. Therefore, our results provide new evidence that Rhus is a novel biflavonoid that regulates osteoclastogenesis.

TRAP is widely used as a cytochemical marker for identifying osteoclasts in bone biology [29]. The expression in osteoclasts is regulated by RANKL, a potent activator of osteoclast differentiation, and significantly elevated when mature osteoclasts engage in bone resorption [30,31]. In this study, we found that Rhus inhibited the TRAP-positive multinucleation of osteoclasts during RANKL-induced osteoclastogenesis. To adhere to the bone surface and resorb mineralized bone, osteoclasts reorganize their actin cytoskeleton to form F-actin rings [29,32,33]. F-actin ring formation during RANKL-induced osteoclastogenesis represents a unique cytoskeletal structure that delineates bone resorption regions known as the sealing zone [34,35]. We also demonstrated that Rhus reduced both the size and number of F-actin rings formed by osteoclasts during RANKL-induced osteoclastogenesis. Increased osteoblast activity and bone formation led to moderate osteoporosis in transgenic mice overexpressing TRAP [36]. TRAP^−/−^ mice exhibit decreased osteoclast activity, including femur deformity, cortical bone shortening, and epiphyseal growth plate [37]. The actin rings of osteoclasts are dynamic structures that undergo production and resorption cycles [38]. Compared to non-resorbing osteoclasts, bone-resorbing osteoclasts possess larger, thicker, and more dynamic actin rings [39]. Furthermore, the abnormal development of actin rings in osteoclasts reduces their ability to resorb bone, as shown both in vitro and in vivo [40]. Therefore, our results suggested that Rhus inhibited osteoclastogenesis and bone-resorbing osteoclasts in primary cultured BMMs.

An activator protein-1 (AP-1) family transcription factor known as c-Fos is activated in the early stages of osteoclastogenesis [41,42]. RANKL enhances NF-ATc1 activation by inducing c-Fos [43,44]. Activated c-Fos collaborates with NF-ATc1 to enhance NF-ATc1 expression and increase the expression of several osteoclast-specific factors, including cathepsin K, TRAP, and chloride [44]. Consistent with this finding, Rhus suppressed RANKL-induced c-Fos and NF-ATc1 expression, thus generating TRAP-positive multinucleated osteoclasts and forming actin rings. NF-ATc1 and c-Fos are overexpressed when the AKT signaling pathway is activated by the RANKL–RANK interaction. Furthermore, this signaling pathway is essential for F-actin generation by osteoclasts [45,46]. In pharmacological studies, RANKL-induced osteoclastogenesis was inhibited by blocking AKT phosphorylation [47,48]. In this study, Rhus significantly inhibited AKT phosphorylation and reduced the expression of c-Fos and TRAP by suppressing the RANKL-induced AKT signaling pathways. In more detail, the effect of Rhus on AKT was modulated by LY294002, indicating that Rhus acts upstream of AKT. Additionally, we demonstrated its effectiveness on MAPKs and NF-κB, apart from AKT. Future studies are essential to broaden the scope of kinome screening and identify additional targets. Furthermore, obtaining vital information for predicting toxicity through gene expression analysis of complex intracellular pathways is crucial. Osteoporosis transcriptional profiles enhance our understanding of bone formation and function as well as identify transcription factors and other mechanisms regulating osteoclast differentiation [49,50,51]. AKT induces the expression of genes associated with osteoclastogenesis, including c-Fos and NF-ATc1, by regulating the GSK3β signaling cascade [52]. Overall, these results suggested that AKT signaling is a crucial target regulated by Rhus during osteoclast differentiation.

In conclusion, our findings demonstrate for the first time that Rhus inhibits RANKL-induced osteoclastogenesis by blocking AKT pathway activation. This inhibition ultimately suppresses the production of key regulators of osteoclast differentiation and function. AKT has a multifaceted function in the pathophysiology of health and disease, extending beyond bone health [53,54,55,56,57,58]. Numerous clinical trials have investigated AKT inhibitors primarily in combination with other medications. In this study, we utilized concentrations of 1 µM or higher. Since many natural compounds are employed without in vivo toxicity at elevated concentrations, we anticipated no stability issues for using Rhus. However, further studies are required to accurately verify its in vivo bioavailability and achievable concentrations. Therefore, Rhus may positively impact bone health and serve as a novel osteoclastogenesis inhibitor that can be used to treat bone diseases.

## 4. Materials and Methods

### 4.1. Plant Material

*Rhus succedanea* was obtained from the market for commercial herbal medicines. The Natural Products Bank of the National Institute for Korean Medicine Development (NIKOM, Gyeongsan, Republic of Korea) has deposited a voucher specimen (P022).

### 4.2. General Experimental Procedures

Column chromatography was performed using Sephadex LH-20 gel (GE Healthcare, Uppsala, Sweden) and silica gel (Merck, Darmstadt, Germany). NMR was conducted on a JEOL ECX-500 spectrometer (JEOL Ltd., Tokyo, Japan) operating at 500 and 125 MHz for ^1^H and ^13^C NMR spectra, respectively. The HPLC were performed using Agilent 1260 series system (Agilent Technologies, Santa Clara, CA, USA) with photodiode array (PDA) and evaporative light scattering detector (ELSD).

### 4.3. Extraction from Dried Rhus succedanea Roots

Dried *Rhus succedanea* roots (6 kg) were extracted three times with 80% MeOH at room temperature (32 L, 3 times). The crude extract (1.3 kg) was suspended in distilled water and partitioned using ethyl acetate (EtOAc) and n-BuOH. The EtOAc-soluble fraction (150.0 g) was subjected to silica gel column chromatography and eluted using a gradient solvent system of 10:1–1:1 (*v*/*v*) n-hexane: EtOAc and 6:1 (*v*/*v*) CHCl_3_–MeOH to yield 41 fractions (RSE-1–41). RSE-15 (1.2 g) was subjected to column chromatography using a Sephadex LH-20 column and eluted with 70% methanol to obtain the active compound (195 mg), which was identified as Rhusflavone (Rhus) by comparing the spectroscopic data with those revealed in previous reports.

### 4.4. Live Subject Statement

All experiments were performed according to the university guidelines and approved by the Institutional Animal Care and Use Committee of Kyung Hee University (KHSASP-23–294).

### 4.5. Primary BMM Culture and Osteoclast Differentiation

Primary mouse BMMs were isolated from 4-week-old female mice as described previously [59]. Briefly, bone marrow cells were isolated from mouse tibiae and femurs by flushing the bone marrow with α-modified Eagle medium (α-MEM; WELGEM, Inc., Seoul, Republic of Korea) and incubated overnight on culture dishes in α-MEM containing 10% fetal bovine serum (FBS, Thermo Fisher Scientific, Waltham, MA, USA) and 1× Gibco antibiotic–antimycotic (Thermo Fisher Scientific, Waltham, MA, USA) in a humidified CO_2_ incubator at 37 °C, with an atmosphere of 95% air and 5% CO_2_. The next day, floating cells were cultured in α-MEM containing 10% FBS, 1× Gibco antibiotic–antimycotic, and 30 ng/mL mouse M-CSF (PeproTech, Cranbury, NJ, USA). Cells with >95% purity that differentiated due to M-CSF stimulation and adhered to the culture dish were classified as BMMs [60]. Adherent BMMs were differentiated into osteoclasts using 100 ng/mL mouse RANKL (PeproTech, Cranbury, NJ, USA), 30 ng/mL mouse M-CSF, and the indicated Rhus concentrations.

### 4.6. Cell Viability

The viability of Rhus-treated BMMs and osteoclasts was analyzed using an MTT assay, as described in previous studies [61]. MTT solution (Sigma-Aldrich, St. Louis, MO, USA) was added to each well and incubated for 2 h. The medium was replaced with 100% dimethyl sulfoxide (DMSO) solution (Sigma-Aldrich) to solubilize the formazan crystals, and the foil-wrapped plates were incubated on an orbital shaker for 15 min. The absorbance of the plates was measured at 540 nm using a Multiskan GO Microplate Spectrophotometer (Thermo Fisher Scientific, Waltham, MA, USA).

### 4.7. TRAP Staining

BMMs were differentiated into osteoclasts using 100 ng/mL mouse RANKL, 30 ng/mL mouse M-CSF, and indicated Rhus concentrations for 5 days. The cells were fixed with 10% formalin for 15 min, washed with 1× PBS, and stained for TRAP following the manufacturer’s instructions (Takara Bio Inc., Shiga, Japan). TRAP-MNCs were measured using an Olympus CKX53 inverted microscope (Olympus Corporation) as described in previous studies [33].

### 4.8. F-Actin-Ring Formation Staining

BMMs were differentiated into osteoclasts using 100 ng/mL mouse RANKL, 30 ng/mL mouse M-CSF, and indicated Rhus concentrations for 5 days. After being permeabilized with 0.1% Triton X-100 and fixed with 4% formaldehyde, the cells were treated for 30 min with TRITC-phalloidin (Invitrogen, Carlsbad, CA, USA). Following a wash with 1× PBS, the cells were stained for 10 min using a 1 μg/mL solution of 6-diamidino-2-phenylindole (DAPI) (Sigma-Aldrich, St. Louis, MO, USA). Images were captured using an intravital multiphoton microscope system (KJ316; Leica Microsystems, Wetzlar, Germany) and an Olympus IX73 inverted microscope (Olympus Corporation, Tokyo, Japan).

### 4.9. Western Blot Analysis

Western blot analysis was carried out following previously established protocols [62]. Briefly, Bradford reagent (Bio-Rad Laboratories Inc., Hercules, CA, USA) was used to quantify equal amounts of protein (20 µg), which were subsequently resolved using sodium dodecyl sulfate–polyacrylamide gel electrophoresis (Bio-Rad Laboratories) and transferred to polyvinylidene fluoride membranes (Millipore, Bedford, MA, USA). The membrane was then blocked with 5% skim milk for 1 h at room temperature and incubated overnight at 4 °C with the primary antibodies. After washing, the membranes were incubated for 1 h at room temperature with secondary antibodies conjugated to horseradish peroxidase (HRP) at a dilution of 1:10,000 (Jackson ImmunoResearch, West Grove, PA, USA). Following washing, the membranes were treated with an enhanced chemiluminescence (ECL) solution (Millipore, Bedford, MA, USA), and immunoreactivity was detected using ChemiDoc Imaging Systems (Bio-Rad Laboratories Inc., Hercules, CA, USA).

### 4.10. Immunocytochemistry

Immunofluorescence was performed as described in previous studies [63]. Briefly, cells were seeded onto 8-well chamber slides (Thermo Fisher Scientific, Waltham, MA, USA), fixed with 10% formalin solution, and permeabilized with 0.1% Triton X-100 solution. After blocking with 3% BSA blocking solution for 1 h at room temperature, the cells were immunostained with anti-c-Fos (1:400, Cell Signaling Technology, Beverly, MA, USA) antibody for overnight at 4 °C. After washing with 1× PBS, the cells were incubated with Alexa Fluor 488-conjugated secondary antibodies (1:500, Invitrogen, Carlsbad, CA, USA) for 1 h at room temperature, washed with 1× PBS, and stained with 1 μg/mL DAPI solution (Sigma-Aldrich, St. Louis, MO, USA) for 10 min. Images were captured using an intravital multiphoton microscope system (KJ316; Leica Microsystems, Wetzlar, Germany) and an Olympus IX73 inverted microscope (Olympus Corporation, Tokyo, Japan).

### 4.11. Statistical Analysis

For statistical analysis, we utilized GraphPad Prism software version 5 (GraphPad Software, Inc., San Diego, CA, USA). All values are represented as the mean ± standard deviation (SD). To determine statistical significance, we employed Dunnett’s post hoc test and one-way analysis of variance. The threshold for statistical significance was set at *p* < 0.05.

## Data Availability

The data generated during the current study are available from the corresponding author on reasonable request.

## Abbreviations

AP-1	Activator protein-1
BMMs	Bone marrow-derived macrophages
HPLC	High-performance liquid chromatography
MAPK	Mitogen-activated protein kinase
M-CSF	Macrophage colony-stimulating factor
MNCs	Multinucleated osteoclasts
NF-ATc1	Nuclear factor of activated T-cells cytoplasmic 1
NMR	Nuclear magnetic resonance
RANKL	Receptor activator of NF-κB ligand
Rhus	Rhusflavone
TNF	Tumor necrosis factor
TRAP	Tartrate-resistant acid phosphatase
