# Correction: Yun et al. Rhusflavone Modulates Osteoclastogenesis Through RANKL-Induced AKT Signaling in Bone Marrow-Derived Macrophages. *Int. J. Mol. Sci.* 2025, *26*, 3025

**DOI:** 10.3390/ijms26157354

**Published:** 2025-07-30

**Authors:** Hyung-Mun Yun, Bomi Kim, Eonmi Kim, Kyung-Ran Park

**Affiliations:** 1 Department of Oral and Maxillofacial Pathology, School of Dentistry, Kyung Hee University, Seoul 02447, Republic of Korea; 2 National Institute for Korean Medicine Development, Gyeongsan 38540, Republic of Korea; bom0203@nikom.or.kr (B.K.); minnie60@nikom.or.kr (E.K.); 3 Honam Regional Center, Korea Basic Science Institute (KBSI), Gwangju 61751, Republic of Korea

In the original publication [1], there was a mistake in Figure 5B as published. The first image in Figure 5B was processed incorrectly, including the scale bar and included in error. The corrected Figure 5B appears below. The authors state that the scientific conclusions are unaffected. This correction was approved by the Academic Editor. The original publication has also been updated.

## Figures and Tables

**Figure 5:**
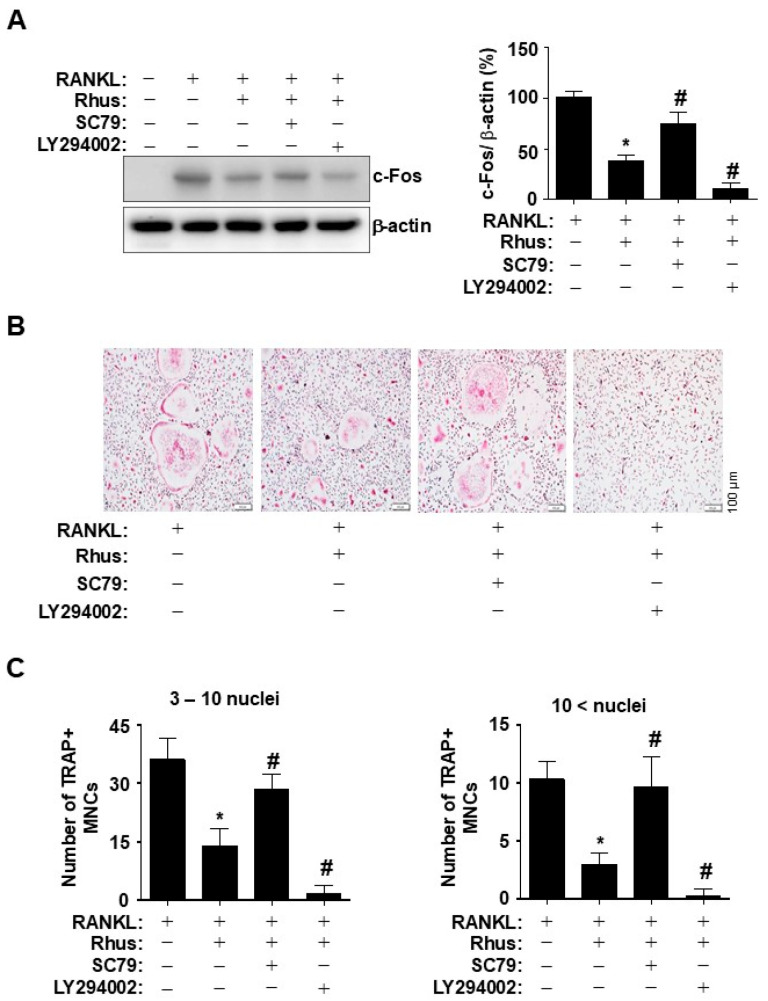
Effects of Rhus on the inhibition of receptor activator of nuclear factor kappa B ligand (RANKL)-induced AKT signaling pathway in osteoclastogenesis. (**A**) Bone marrow macrophages (BMMs) were differentiated with 10 μM Rhus, in the presence or absence of 1 μM SC79 or LY294002, for 1 day. Western blot analysis was used to assess c-Fos and β-actin expression levels. The relative level (%) is presented as a bar graph. (**B**,**C**) The BMMs were cultured in 30 ng/mL M-CSF and 100 ng/mL RANKL with 10 μM Rhus, in the presence or absence of 1 μM SC79 or LY294002, for 5 days. Mature osteoclasts were detected with tartrate-resistant acid phosphatase (TRAP) staining (pink) (**B**). TRAP-positive multinucleated osteoclasts (MNCs) were counted under a microscope (**C**). A quantity of 3–10 nuclei (**left**), 10 < nuclei (**right**). Scale bar: 100 μm. *, *p* < 0.05 compared with RANKL alone. #, *p* < 0.05 compared with RANKL + Rhus. The data presented are derived from three separate experiments and are expressed as the mean ± standard deviation (SD).
